# Bioengineering Skin Substitutes for Wound Management—Perspectives and Challenges

**DOI:** 10.3390/ijms25073702

**Published:** 2024-03-26

**Authors:** Karolina Kondej, Małgorzata Zawrzykraj, Katarzyna Czerwiec, Milena Deptuła, Agata Tymińska, Michał Pikuła

**Affiliations:** 1 Department of Plastic Surgery, Medical University of Gdansk, 80-214 Gdansk, Poland; karolina.kondej@gumed.edu.pl; 2 Department of Clinical Anatomy, Medical University of Gdansk, 80-211 Gdansk, Poland; malgorzata.zawrzykraj@gumed.edu.pl (M.Z.); katarzyna.czerwiec@gumed.edu.pl (K.C.); 3 Laboratory of Tissue Engineering and Regenerative Medicine, Department of Embryology, Medical University of Gdansk, 80-211 Gdansk, Poland; milena.deptula@gumed.edu.pl (M.D.); agata.tyminska@gumed.edu.pl (A.T.)

**Keywords:** wound healing, chronic wounds, hydrogels, scaffolds, regenerative medicine, tissue engineering, stem cells, 3D bioprinting

## Abstract

Non-healing wounds and skin losses constitute significant challenges for modern medicine and pharmacology. Conventional methods of wound treatment are effective in basic healthcare; however, they are insufficient in managing chronic wound and large skin defects, so novel, alternative methods of therapy are sought. Among the potentially innovative procedures, the use of skin substitutes may be a promising therapeutic method. Skin substitutes are a heterogeneous group of materials that are used to heal and close wounds and temporarily or permanently fulfill the functions of the skin. Classification can be based on the structure or type (biological and synthetic). Simple constructs (class I) have been widely researched over the years, and can be used in burns and ulcers. More complex substitutes (class II and III) are still studied, but these may be utilized in patients with deep skin defects. In addition, 3D bioprinting is a rapidly developing method used to create advanced skin constructs and their appendages. The aforementioned therapies represent an opportunity for treating patients with diabetic foot ulcers or deep skin burns. Despite these significant developments, further clinical trials are needed to allow the use skin substitutes in the personalized treatment of chronic wounds.

## 1. Introduction

The healing process consists of four main phases: hemostasis, inflammation, proliferation and remodeling [1]. The first phase, hemostasis, is activated immediately after the tissue damage occurs. Platelets are activated, and they transmit a chemical signal to form blood clots. At the same time, epidermal growth factor, platelet-derived growth factor, transforming growth factor beta and chemokines are released. Thromboxane A2 forms at this time from prostaglandin A2 under the influence of thromboxane synthase, which strongly activates platelets, constricts blood vessels and plays a role in recruiting macrophages, neutrophils and endothelial cells [2].

In the inflammatory phase, leukocytes and thrombocytes release more cytokines. Moreover, serotonin and histamine are released from thrombocytes, causing an increase in cellular permeability. Platelet-derived growth factor stimulates the migration of fibroblasts, and together with TGF-beta, enhances their division and multiplication. In turn, neutrophils, monocytes and endothelial cells adhere to the fibrin scaffold. Neutrophils are responsible for phagocytosis of cellular debris and bacteria, thus enabling wound disinfection. Monocytes differentiate into macrophages. Neutrophils, monocytes and macrophages cause the release of pro-inflammatory cytokines IL-1, IL-6, IL-8 and TNF-alpha, as well as growth factors platelet-derived growth factor, transforming growth factor alpha, transforming growth factor beta, fibroblast growth factor and insulin-like growth factor [3].

The proliferative phase is characterized by the migration of fibroblasts to the site of damage. Their task is to produce collagen. Fibroblasts, under the influence of signaling factors originating from platelets, endothelial cells and macrophages (TGF-beta, PDGF) produce extracellular matrix proteins or are involved in the differentiation into myofibroblasts, which results in wound closure. Fibroblasts degrade the temporary matrix, producing metalloproteinases, thereby replacing the temporary matrix with granulation tissue, which is characterized by a high content of fibronectin, immature collagens and proteoglycans [4]. The resulting granulation tissue forms a scaffold for wound migration and scaffolding, thereby promoting the formation of new blood vessels as well as the deposition of mature ECM. To meet the high metabolic demands of a healing wound, new blood vessels are formed. Angiogenesis is induced by hypoxia, which triggers the expression of hypoxia-inducible factors (HIF), cyclooxygenase II and, above all, the release of vascular endothelial growth factor. In response to these changes, endothelial cells proliferate and migrate to the wound bed. In this phase, intense migration of keratinocytes occurs, driven by metalloproteinase-1 and 9 and plasmin, which enables the degradation of the temporary wound bed [5].

During the remodeling phase, the extracellular matrix transforms into mature scar. Type III collagen is replaced by type I collagen. The number of new blood vessels is reduced. The main goal of this phase is to obtain maximum tensile strength, which is obtained by reorganization, degradation and resynthesis of the extracellular matrix. An attempt is made to restore healthy tissue. As a monolayer of keratinocytes covers the surface of the wound, their migration is halted and new, layered epidermis is rebuilt. As the wound closes, type III collagen degrades, and thus collagen I synthesis increases [6].

The healing process is influenced by a myriad of exogenous and endogenous factors. Diabetes, immunosuppression, corticosteroids and smoking may complicate wound healing [7]. Patients with diabetes, in particular, often exhibit abnormalities in cells characterized by low proliferation potential and an impaired ability to respond to environmental signals [8]. A characteristic feature of every wound, regardless of its origin, is chronic inflammation of the wound bed and lack of healing [9]. Due to certain abnormalities in the healing process, endothelial cells may disproportionately facilitate the expression of vascular cell adhesion molecule 1 and interstitial cell adhesion molecule 1, which is driven by infection and cell extravasation, thereby resulting in excessive recruitment of inflammatory cells to the wound bed. The accumulation of inflammatory cells in the wound bed causes the production of reactive oxygen species, which damage the extracellular matrix and structural elements of the cell membrane, thus leading to premature cell aging. Reactive oxygen species and pro-inflammatory cytokines induce the production of serine proteinases and metalloproteinases, which are responsible for the destruction and inactivation of the extracellular matrix and growth factors needed for the proper functioning of the cell. Inactivation of proteinase inhibitors through proteolytic degradation enhances this process. In chronic wounds, an increase in the production of growth factors is observed, but their quantity and bioavailability are significantly reduced [10]. Factors with high levels in pathological wounds are summarized in Table 1.

Chronic wounds can be treated through standard methods such as dressings, negative pressure therapy or transplantation [8,11,25]. Transplantation of skin on healthy MHC-mismatched recipients leads to rapid immune rejection within 10–14 days with characteristic skin tissue destruction. Allogeneic skin grafts are rejected in approximately 20–30 days, even if grafts are placed over the fully excised burn wound base. The skin grafts placed in the wound must establish their own blood vascularization [26]. As a result of the aforementioned host immune reaction and possibility of rejection of skin transplants, alternative methods for wound management are actively being explored [27].

Bioengineered skin substitutes not only serve as protective dressings, but also have the potential to contribute to tissue regeneration. Their primary task is to reduce the number of bacteria penetrating and colonizing the wound and to reduce fluid loss. Covering the wound with a skin substitute causes the inflow of fibroblasts, keratinocytes, endothelial cells, neutrophils and macrophages into the wound bed. Subsequently, these cells secrete cytokines and growth factors that stimulate the processes of angiogenesis, extracellular matrix deposition and re-epithelialization [28].

## 2. Wound Healing Complications—Clinical Need for Advanced Products

**Figure 1:**
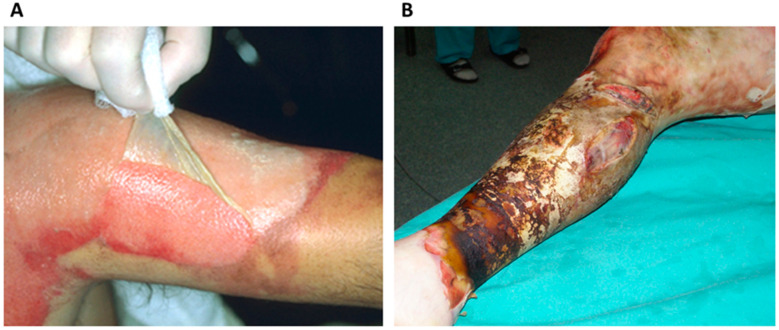
Examples of burn wounds in patients. (**A**)—Second degree burn, i.e., intermediate skin thickness, the epidermis with part of the dermis that has been destroyed separates from the living layer in the form of a blister; (**B**)—Deep burn—all skin, waxy and black tissue are destroyed; this is necrosis. A circumferential burn threatens the build-up of pressure inside the limb and may cause blood vessels to occlude, leading to ischemia of the entire limb. Compressive cuts are made to maintain blood flow in the blood vessels (Dpt. of Plastic Surgery, MUG, Poland).

Experts expect the wound care market to be valued at USD 23.14 billion in 2024 and grow to USD 29.57 billion by 2030. In 2023, this market was valued at USD 22.25 billion [29]. The incidence of chronic wounds is closely related to the occurrence of chronic diseases: diabetes, cardiovascular diseases, obesity and autoimmune diseases. Factors contributing to the development of chronic wounds include an aging population, lifestyle diseases and the challenges associated with caring for immobile patients who are confined to beds. The inability of affected people to adjust their position in bed results in local tissue necrosis due to local ischemia from prolonged pressure. Moreover, burns and trauma cases such as accidents and surgeries frequently result in complex wounds. Examples of extensive burn wounds from our center are shown in Figure 1. Appropriate care of a chronic wound is extremely important because it can easily lead to bacterial colonization, which will make wound treatment more difficult. As a consequence, neglect or incorrect treatment may lead to amputation. Additionally, it is important to emphasize that improper wound healing can lead to the formation of keloids and hypertrophic scars, which pose significant challenges for physicians.

It is also worth mentioning that the COVID-19 pandemic placed a strain on the healthcare system [30]. With the increase in the number of confirmed cases and the requirement to maintain social distancing, it became a challenge to conduct doctors’ visits or have wounds cleansed by specialists. The more frequent visits for various types of wounds, the better treatment results can be achieved. Lack of regular visits to a wound care clinic can increase the number of hospitalizations twenty-fold [31]. The pandemic has shown how important patient education is. The implementation of telemedicine and the transfer of medical care from the clinic to the patient’s home during the pandemic allowed for risk reductions in viral infections in medical facilities, thus increasing patient safety [32].

Taking these factors into account, the treatment of difficult-to-heal wounds becomes a very serious clinical, social and economic challenge. Well-selected, effective therapy following the doctor’s recommendations allows you to restore function and improve aesthetics, making it possible to return to full fitness. Therefore, research teams from all over the world are looking for more effective solutions and treatment methods. With the development of tissue engineering and regenerative medicine, many solutions personalized to specific patients have been developed, ranging from dressings that ensure the release of drugs controlled in time and space, three-dimensional printed biomaterials, to advanced skin substitutes that allow the replacement and regeneration of entire tissue layers [33]. Trends prevailing in the biological skin substitutes market predict that it will reach USD 321 million by 2023 and lead to the development of more sophisticated solutions [34]. Due to regulatory constraints, high costs and scientific challenges, there is a need to produce and improve advanced skin substitutes and artificial skin models that will lead to lower costs. Automatic bioreactors with automation and standardization, or 3D bioprinting combined with compatible bioinks are achievements in the field of tissue engineering [35]. It is hoped that thanks to this, in the future there will be no need for autografts, which may leave visible scars on the body as well as increase the risk of bacterial infections when performing this procedure.

The importance of chronic wounds is overshadowed by their causes. It is a silent epidemic that affects the quality of life of over 40 million people around the world [36].

## 3. Skin Substitutes—Characteristics and Clinical Application

Skin substitutes are one type of tool that has been shown to be effective in healing wounds, and have been actively explored in the process stimulating wound healing. Skin substitutes are a heterogeneous group of biologic, synthetic or biosynthetic substances that are used to heal and close wounds and temporarily or permanently fulfil the functions of skin [37,38,39,40].

Kumar P. and Gupta A [41] divided the skin substitute materials into the following three classes according to their structure:

Class I—one- or two-layer temporary dressing materials;
Class II—single-layer skin substitutes (epidermal or dermal);
Class III—complex skin substitutes (dermal–epidermal).

**Figure 2:**
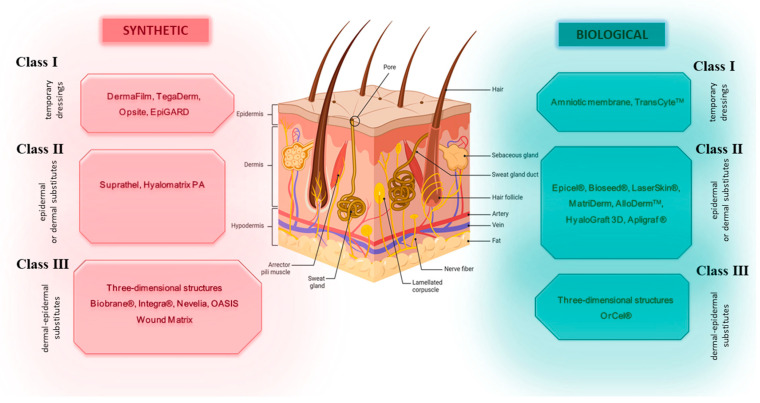
Classification of skin substitutes depending on structure and type of biomaterial used. Created with BioRender.com (accessed on 19 February 2024).

Depending on the type of biomaterial used in the construction, substitutes are divided into biological or synthetic (biodegradable, non-biodegradable). Moreover, the cells used in substitutes can be autologous, allogenous or xenogenous. The advantage of biological substitutes is the relatively intact structure of the extracellular matrix. The advantage of synthetic skin substitute materials is the possibility of modifying their properties, e.g., enrichment with growth factors and elements of the extracellular matrix. However, a notable drawback of synthetic substitutes is their absence of a basement membrane [42,43]. Due to their ability to be incorporated into the tissue, there are temporary, semi-permanent and permanent substitutes. Another division is into cell-based and cell-free substitutes. When biomaterials are pre-seeded or have cells embedded in their matrix, they are classified as cellular artificial skin grafts [37,44,45,46]. A classification of skin substitutes in shown in Figure 2.

The role of temporary skin substitutes is to close the wound and provide protection against secondary mechanical injuries and bacterial colonization; moreover, they prevent evaporation. Temporary skin substitutes are used in clinical practice, both to cover superficial wounds (then, they are used until secondary epithelization is completed) and in the treatment of deep wounds (before planned skin transplantation). They can be used to protect the sites from which the skin graft has been removed (donor site). Furthermore, these skin substitutes stimulate epithelialization and reduce pain [43].

### 3.1. Class I Skin Substitutes

Class I skin substitutes do not contain cells. They are composed of one or two layers of dressing. They improve the mechanical properties of the skin; however, they do not improve the biological properties of the cells [47].

TransCyte^TC^—known as Dermagraft^TC^—is a two-layer dressing, used for second- and third-degree burn wounds. Dermagraft was approved by the FDA in 1997. It consists of extracellular matrix, type I collagen, growth factors, proteoglycans and fibronectin synthesized by neonatal human dermal fibroblasts that have previously been seeded on nylon mesh covered with porcine collagen. The product is non-biodegradable and is only for temporary use, where fibroblasts can be multiplied for about 17 days. Once the matrix is formed, the fibroblasts are removed by freezing (−20 to −70 °C). TransCyte can be used as an allograft for immediate application to a fresh wound, or it can be frozen [48]. Amani et al. studied the effectiveness of TransCyte dressings in patients with partial-thickness burns. In their research, 92 patients were treated with TransCyte with dermabrasions, while 18 patients were additionally treated with an intermediate-thickness skin grafts. The length of hospital stay was shown to be shorter with the aforementioned treatments compared to standard therapy. These results correlate with faster and more effective treatment with skin substitutes [49]. The effectiveness of TransCyte was compared with other preparations used in wound treatment—BioBrane and Silvazine cream. The epithelialization period was the shortest when using TransCyte and amounted to 7.5 days. Also, the number of defects requiring autografts in this group was the lowest and amounted to 1 in 20 patients [50].

DermoFilm^®^ can be used as a spray or single-layer patch. It is a hydrocolloid skin substitute used on skin graft wounds, bedsores, closed surgical wounds and superficial ulcers [51]. DermoFilm has not been widely described in publications. See et al. tested the efficacy of this skin substitute in 49 patients with skin lesions after radiotherapy. All of the patients noted improvement after using the dressing and no adverse effects [52].

Amniotic membrane (AM) is an inner layer of the amniotic sac, which completely surrounds the embryo and covers the inner part of the placenta [53]. It consists of two layers, amnion and chorion. Amnion consists of epithelial cells and collagen-rich tissue, while chorion is mostly built of collagen and elastic fibers, as well as proteoglycans [54]. It is characterized by some features that enable its use as wound healing material. These include the presence of growth factors, e.g., epidermal growth factor (EGF) and keratinocyte growth factor (KGF), which provide protection against bacterial infections and prevention of water loss [55]. Also, cellular and acellular forms of AM are reported to show anti-inflammatory properties. The first published reports of fresh AM use in wound healing date back to 1910, but it has also been applied in medicine since 600 BC [56]. AM has been used as wound dressing for burns, cornea injuries, neuropathic wounds, as well as in tissue reconstruction in the oral cavity or bladder [57]. A meta-analysis of the use of AM products in the treatment of diabetic foot ulcers (DFUs) summarizing the results of five randomized controlled trials (259 patients receiving amniotic tissue products or standard of care) showed increased rates of wound healing in these patients [58]. For example, one of these studies evaluated the effect of Graphix, a cryopreserved AM product, and the results showed reduced healing times (42 days versus 69 in control patients receiving standard care) and significantly lower probabilities of side effects like infections and hospitalization [59]. AM products are also used as wound dressings to increase wound healing in split-thickness skin graft donor sites (STSGDS). A meta-analysis summarizing the results of seven included studies (219 patients) compared the efficacy of AM and other dressing for STSGDS. The analysis confirmed the safety and effectiveness of AM, which, in comparison to the control group, showed significant reduction in healing time with no difference in the infection rate and pain [60]. Another meta-analysis summarized the effect of AM on wound healing in burns and its tolerability. The authors included 11 trials (816 patients with burns) and compared the use of AM with other treatments. The analysis showed that compared to conventional methods, AM is more effective, reducing healing time, bacterial infections and decreasing the number of wound dressing changes. However, it was less effective than honey. No AM-related adverse effects were observed [61].

Opsite is polyurethane membrane. Its main features include providing occlusion, adherence to the wound and sterility. The first trials of Opsite’s use were described in 1975, where the dressing was used in 53 people at their skin graft sites. Reduced pain and faster healing were described as advantages of using the polyurethane film. However, a lack of postoperative fluid absorption properties was noted [62]. Subsequent trials were conducted in 1985, among others. These involved 150 people with partial-thickness burns, where wounds were present in various anatomical locations. Treatment with Opsite was successful in 80% of cases [63]. Various modifications of the abovementioned dressing were utilized. In 2007, Wilde and Loudon showed modified Opsite as a vacuum pack. It was used in surgery patients after laparostomy. The dressing was applied in 11 patients with intra-abdominal hemorrhage, peritonitis or intestinal blood flow dysfunction. Treatment ended with closure of the abdominal fascia in 10 patients, as late as 8 days after reconstruction [64]. Additionally, in 2017, the clinical trial numbered NCT03190447 began. It compares the efficacy of treatment with various dressings, including Opsite, to traditional povidone-iodine-soaked gauze dressings in patients with burns. Opsite Post-Op Visible is a hydrocellular foam dressing that is waterproof and transparent. The results of the trial will include skin integrity, healing time, incidence of side effects such as exudates, erosions, blisters and patient satisfaction [65].

Tegaderm™ (3M) is a transparent foil dressing. This dressing was introduced to the market in the United States in 1981 by 3M. Six years later, the company became the leader in market share for foil dressings in the US [66]. The dressing provides a waterproof barrier that protects against external contamination: mainly bacteria, viruses (diameter 27 nm or larger), but also external fluids, blood and body fluids. Tegaderm™ is available on the market in a non-sterile version (roll dressing, for self-cutting in various widths) and sterile version (with a frame that makes application easy, reduces the risk of the dressing sticking to gloves, and is available in various shapes and sizes) [67]. A series of Tegaderm™ line dressings can be kept for up to 7 days; the breathable foil allows oxygen and steam to pass through. The dressing adapts to the skin, which allows the patient to move freely, and the transparency of the dressing allows for constant monitoring of the injection site. A big advantage is the ability of not having to remove this dressing during radiological examinations; it can be left on while receiving radiotherapy. Tegaderm™ is used primarily as skin protection (e.g., during negative pressure wound therapy), more stable support for main dressings, fixation of medical devices and infusion drains [68]. Tegaderm™ I.V. stabilizes the catheter, minimizing its movements. The dressing is intended to protect the insertion sites of vascular catheters, epidural catheters, pulmonary artery catheters, dialysis catheters, insulin pumps, central catheters (excluding subclavian, jugular, femoral veins, central catheters led from peripheral access), feeding tubes and butterfly needles. Tegaderm™ Diamond dressings are used to protect vascular puncture sites. This dressing has a high-water vapor permeability coefficient. It works well in hot, humid conditions or for patients with excessive sweating. It can also be used on closed intact surgical wounds, abrasions, skin tears and blisters. The Tegaderm™ line includes a transparent foil dressing with a window without glue for attaching vascular punctures, which reduces the risk of the dressing sticking to the needle when removed. It can be used with implanted venous ports, Huber needles, which do not cause tissue loss, and with implanted venous ports in oncology and hematology patients [69]. Since the 1980s, Tegaderm has been subjected to research according to pubmed.ncbi.nlm.nih.gov [70]. Forty years later, it is also present in randomized trials for various applications [71,72]. In a randomized study on the control of surgical site infections, the effectiveness and safety of the Trushield NXT dressing (Healthium Medtech Limited, Bangalore, India) was compared with the traditional Tegaderm HP+ Pad foil dressing with a non-adherent pad (3M, Bangalore, India) for the dressing of wounds after obstetric and gynecological procedures. In the final summary, both dressings performed well. Tegaderm was appreciated for its flexibility and ease of use. Patient satisfaction with the use and properties of the dressings was similar in both cases [71].

EpiGARD cleanses the wound and at the same time maintains a moist environment that optimally prepares the wound for subsequent skin transplants. Its upper surface consists of a thin, microporous foil that is permeable to air and moisture. This prevents the accumulation of secretions while protecting the wound from bacteria and other external factors. The bottom layer forms a working matrix made of flexible, soft polyurethane. The wound exudate adheres to a fine porous structure and is removed with necrotic tissue after dressing changes. Fibroblasts and vessels begin to grow in the area of the wound and open the polyurethane foam [73,74]. Viscardi et al. described a reconstruction within the arm of a 67-year-old patient with an extensive melanoma lesion. Initially, a ganglion flap graft was performed, and then the site was covered with EpiGARD. The following week, the wound was reduced in size and re-covered with the substitute. Complete closure was performed after 2 weeks. No side effects were shown for the applied treatment. The cosmetic effect was assessed as satisfactory at 8 months after treatment [75]. In contrast, Grassner et al. used EpiGard in nine patients after craniectomy. The researchers estimated that complete wound closure was possible with 20 changes of the substitute. Cerebrospinal fluid leakage was noted in two patients. Other complications included infections of both the site of injury and the central nervous system [74].

### 3.2. Class II Skin Substitutes

The primary role of epidermal substitutes is to support the reconstruction of the epithelium and cover the wound bed, as well as protect against water and protein loss. They also provide a barrier to pathogens that cause infections. Modern epidermal grafts are usually in the form of a thin film that promotes adhesion and keratinocyte proliferation. Epidermal skin substitutes typically use cultured keratinocyte autografts [47].

Epicel (Vericel Corporation) is one of the oldest skin substitutes. It was produced in 1975 and entered the market in 1988. It is based on in vitro cultured keratinocytes that are transferred to non-stick gauze, and then applied to the wound. Its advantages include permanent and fast wound closure, pain relief, small donor sites (secondary wounds) and better functional and aesthetic results compared to autologous skin grafts. The disadvantages include the necessity to collect a fragment of the skin, long waiting times for the keratinocyte culture (meanwhile keeping the patient in good condition), high cost, and unstable attachment of keratinocytes to the substrate without the basal layer. The indications for its use are burns, chronic leg ulcers and blistering epidermis [76]. In 2000, a study related to the use of Epicel in 30 patients with burn wounds was published. The dressing provided a high survival rate of about 90%. Recovery of permanent skin coverage occurred in about 26% of patients. Epicel was evaluated to treat extensive burns with severe trauma [77]. Kimia et al. described a case report of a newborn with necrotizing fasciitis of the scalp. The vast majority of the scalp had to be removed during surgery. The defect in the first stage was covered with Integra. This was followed by the application of an Epicel dressing over the altered tissue. The epidermis for Epicel was obtained by skin biopsy in the left groin. Two months after the procedure, the wound was assessed as 90% covered [78].

BioSeed^®^ is a cellular epidermal substitute for the treatment of chronic leg ulcers, obtained by culturing autologous keratinocytes in a fibrin sealant [79]. Johnsen et al. evaluated the effectiveness of the BioSeed substitute in 52 patients with ulcers in the lower leg area. In 29 patients, wound healing occurred for up to 6 weeks. From day 8 to day 42 after surgery, an increase in epithelialization to 62.5% was noticed. No healing was observed in less than 18% of patients [80]. In addition, BioSeed has been used to treat hard-to-heal venous ulcers. Undergoing standard treatment, 24 patients out of 109 achieved complete wound healing. In contrast, in the group with the substitute used, 44 patients out of 116 fully healed. The healing time was estimated at 201 days in the control group and 176 days in the study group [79].

Laserskin^®^ is a thin and transparent epidermal cell transplant for the treatment of full-thickness burn wounds or chronic ulcers, and is made of an esterified benzyl hyaluronan derivative with autologous keratinocytes grown on its surface [81]. Pajrdi et al. used LaserSkin and Hyalograft 3D in patients in whom standard treatment failed to produce the desired results. The LaserSkin substitute was covered with allogeneic keratinocytes, while Hyalograft 3D was covered with fibroblasts; the first was used in patients with superficial wounds, while the second was utilized to treat deep wounds of the lower leg. After the substitutes were placed along with the cells on the wound, a secondary dressing containing silver nanocrystals was applied. No side effects were noticed, and both the wound size and exudate decreased. However, the degree of healing was estimated to be greater with LaserSkin with keratinocytes [82].

Suprathel is a resorable epidermal substitute characterized by high oxygen and water vapor permeability corresponding to the physiological conditions of the natural skin. Suprathel consists of lactocapromer with polylactic acid as the main ingredients. It is completely synthetic and does not contain collagen, so the biological risk is completely eliminated. The time of hydrolytic degradation of the dressing is 4 weeks. It is used for the treatment of deep burns, donor sites after skin grafting, abrasions and covering postoperative wounds [83]. The study by Keck et al. evaluated the treatment given to 18 patients with partial-thickness burns. A skin graft or Suprathel dressing was used as a model of healing. Wound closure after 15 days occurred in six patients with the substitute and in 16 patients with the graft. Three months after treatment, less scarring was observed in areas with Suprathel applied [84]. In addition, Lubna et al. compared the use of paraffin-soaked gauze to Suprathel dressing in skin transplant patients. The pain was rated high in 21 out of 32 patients with the conventional treatment and in 10 out of 32 patients with the skin substitute. The healing time was also rated as significantly faster with Suprathel in most subjects (less than 14 days) [85].

Apligraf is the first and most advanced skin substitute, also called human artificial skin. For clinical use, it was implemented in 1998. The production of this artificial tissue is relatively complicated, even compared with other substitutes. First, fibroblasts collected from the foreskin of a newborn are mixed with bovine type I collagen, and the mixture is then exposed to elevated temperatures to form a loose matrix. The material is left for a period of two weeks when new collagen is formed and a dense network of its fibers is formed in the matrix. Then, the resulting matrix is sown with a suspension of living cells (from the same or a different newborn) and left for a further 4 days for them to proliferate. During the last two days in tissue culture, the concentration of calcium ions increases, which results in the formation of a stratum corneum; thus, the substitute is ready. Although Apligraft is a complex cellular skin substitute, it requires the use of autologous skin grafts. This is related to the short survival of added cell elements, ranging from 1 to 2 months [86]. The use of Apligraf yields excellent results in the treatment of venous ulcers that are not amenable to other therapies, as well as diabetic foot cases. Studies have shown that this skin substitute is more effective than other treatments, with a ratio of 69%:49% when it comes to patients being fully cured within 6 months [87].

Dermal substitutes are easy to use and fairly durable. Their utilization reduces the risk of scarring and contractures. The dressings most often contain fibroblasts. In order to reduce production costs and eliminate certain technical problems, dermal skin substitutes are frequently made of cell-free biomaterials. The primary role of these cell-free dermal constructs is to act as the backbone for the migration and infiltration of fibroblasts and endothelial cells after implantation into a living organism [88].

MatriDerm is a dermal matrix composed of bovine collagen covered with elastin. It was approved for use by the US Food and Drug Administration in 2021. It is designed for full-thickness skin repair in combination with split-thickness skin grafts. The product comes as a graft template in two thicknesses—1 mm and 2 mm. Before use, the substitute should be immersed in saline solution [89]. Jackson et al. used a skin substitute in a 3-year-old girl with full-thickness burns on her face and neck areas. The dressing was applied under a transplanted piece of skin from the thighs for facial reconstruction. After a year of follow-up, the wounds were shown to have healed, and the skin’s functionality and aesthetic qualities were restored. The patient was able to eat and drink normally and fully move her eyeballs and eyelids [90]. Hones et al. used MatriDerm to cover limb wounds characterized by exposure of anatomical structures such as tendons or bones. For 6 wounds out of 11 studied, closure was noted after application of the substitute. Two subjects also underwent subsequent skin grafting. Healing occurred after an average of 49 days [91]. In contrast, Orabona et al. evaluated the effect of MatriDerm on skin defects following surgical removal of head tumors. After tumor excision, a substitute was applied and skin grafting was performed in 16 patients. The wounds healed, and a reduction in scarring, improved blood supply and adequate skin pigmentation were observed during follow-up visits. However, alopecia was observed in patients at the reconstruction site [92].

Alloderm^TM^ was approved for clinical use in 1992 by the FDA. It is a cell-free matrix made directly from fresh skin taken from a cadaver, which is prepared in high-concentration saline to remove the epidermis and bathed in solutions to separate cellular structures. It is then dried, leaving an non-immunogenic functional matrix with an undamaged basement membrane complex and glycosaminoglycans. Wester et al. conducted a study evaluating the effectiveness of using Alloderm with an intermediate-thickness skin graft. They used the aforementioned method to treat wounds in the forearm area in 80 patients. The side effects included infection, incomplete functional capacity, incidence of bleeding or tendon exposure, and were not statistically significant. No re-transplantation of skin was required after treatment [93]. Widmyer et al. evaluated the effectiveness of two types of Alloderm in patients undergoing breast recontouring. One group was treated with freeze-dried Alloderm (FD Alloderm), while the other group was treated with sterile Alloderm. Transplants performed between 2009 and 2016 in 236 patients were estimated. FD Alloderm was shown to cause more infections and reoperations. Considering skin lesions such as hematomas or necrosis, the efficacy of the two groups was similar at no more than 10% [94].

Hyalomatrix PA is a skin substitute that consists of a hyaluronic acid benzyl ester covered with a semi-permeable silicone membrane on top. Hyaluronic acid plays a crucial role in creating a stimulating environment for cell migration, while preserving the extracellular space structure within the skin [95]. After the underlying tissue has healed, usually 14–21 days after application, the silicone layer can be removed. Hyalomatrix can be used on burn wounds, on chronic wounds, and on those that require rapid healing [96]. It has been shown to aid in the healing of postoperative wounds in combination with negative pressure therapy, and works well in combination with platelet-rich plasma gel in the treatment of severe sinus abscesses [88,97]. The main adverse reaction identified in a prospective multicenter study of Hyalomatrix in the treatment of difficult chronic wounds was infection [98]. Gravante et al. analyzed data on 57 burn patients treated with Hyalomatrix PA. Most often, the substitute was applied after cleaning the wound. After 7 days, re-epithelialization was noted, and after 29 days, the wound was completely closed. No side effects were observed for the method of treatment used [99].

Hyalograft 3D is a substitute composed of autologous skin fibroblasts and a scaffold formed of hyaluronic acid. Caravvagi et al. tested the effectiveness of the substitute in patients with diabetic ulcers. The study group of 43 patients was treated with Hyalograft 3D, while the control group (36 patients) was treated with paraffin gauze. After 7–10 days of using the substitute, Laserskin was applied to the wounds. Faster healing of ulcers in the study group was achieved, with an average of 63 days. A higher healing rate was also demonstrated compared to the control group. No side effects specific to either group were observed [100]. Another study using Hyalograft 3D was also based on patients with diabetic ulcers. A foam dressing was used as a control. The group of patients treated with the skin substitute showed a wound closure rate of 84%. The control group had a fully closed wound rate of 34%. The healing time was also estimated to be shorter with the tested substitute. No adverse effects were demonstrated [101].

Permacol™ is a porcine dermal collagen implant intended for hernia and abdominal wall repair, used since 1980s. During the production of the implant, cells, cell debris, DNA and RNA are removed from pig skin, without damaging the three-dimensional collagen matrix. Then, to increase durability, the acellular collagen matrix is cross-linked. The action of hexamethylene diisocyanate (HMDI) increases the degree of cross-linking [102]. This makes it more resistant to the degradative effects of collagenase, while maintaining tissue integration [103]. The prepared product is immediately ready for use and is available in various sizes. J. A. O’brien [104] showed that the use of Permacol in the patient allowed the product to integrate with human elastin and collagen in the remodeling process. The implant used turned out to be durable and, at the same time, the vessels became ingrown without any final inflammation.

### 3.3. Class III—Complex Skin Substitutes (Dermal–Epidermal)

Complex dermal epidermal substitutes are the most advanced of all types of skin substitutes. The dermal and epidermal matrices are often prepared and colonized by culturing keratinocytes in the superficial layers of the skin substitute with embedded fibroblasts in deeper layers. Constructs containing both types of cells enable the migration and proliferation of keratinocytes and fibroblasts by releasing GF and cytokines, which are very important for the rapid healing process and promoting re-epithelialization [97,105]. To produce functional dermo–epidermal constructs, researchers often use various advanced techniques, such as 3D bio-printing [106] or electrospinning [107].

**Figure 3:**
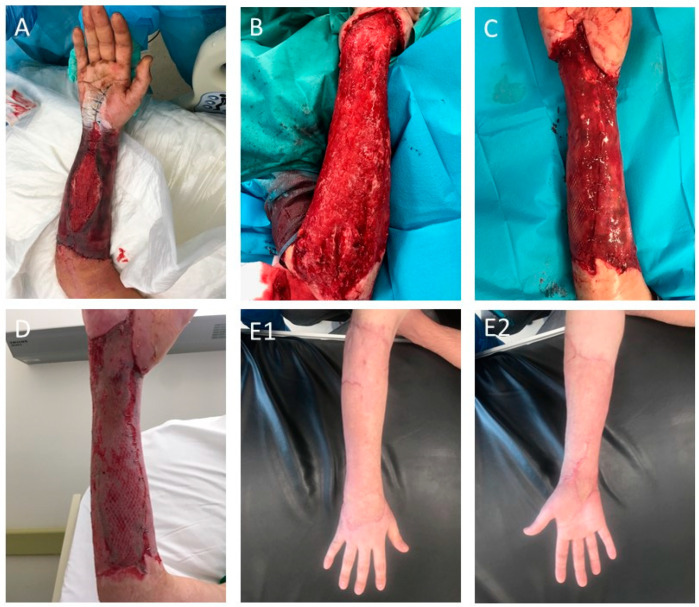
An example of INTEGRA use for wound healing. (**A**)—Necrosis of integuments and circular wound of the right upper limb; (**B**)—The wound after excision of necrotic tissues; (**C**)—STAGE I: Covering of the tissue defect with the INTEGRA matrix for the regeneration of the dermis; (**D**)—STAGE II: Transplantation of the intermediate skin thickness onto the healed INTEGRA matrix; (**E1**,**E2**)—The wound 1 year after the treatment. (Dpt. of Plastic Surgery, MUG, Poland).

The Integra Dermal Regeneration Template provides a matrix for regeneration of human skin. Integra is one the best-known skin substitutes; it is the first product used in the process of burn wounds treatment approved by the FDA. It is a two-layer product; the outer layer is in the form of a silicone membrane, is impermeable to water, prevents fluid loss and protects against mechanical injuries and bacteria. Moreover, it provides immediate wound closure and conditions similar to natural skin conditions. The underside part consists of a three-dimensional matrix made of bovine collagen with the addition of chondroitin sulfate, a shark-derived glycosaminoglycan [108,109]. The porous matrix, which is biodegradable, stimulates cell growth and collagen synthesis. After the wound is covered with Integra, the tissues are revascularized within 3 weeks. After this time, the silicone top layer is replaced with a very thin autologous skin graft. The substitute yields good results, even with large wound areas. However, its disadvantage is the stiffness of the matrix and problems with adherence to the substrate, and thus ingrowth into the patient’s tissues. Additionally, the cost is high and at least two treatments are required [110]. Clinical trials evaluated the potential of Integra in the treatment of non-healing DFUs. In the study, 307 patients with DFUs were subjected into two groups: a control group (153 patients receiving 0.9% sodium chloride gel, secondary dressing and an offloading/protective device) and a treatment group (154 patients). The study showed that the use of Integra decreases healing time (43 days versus 73 days in the control group) and increased wound closure rates. Less adverse effects were also observed [111]. Similarly, Integra showed good wound healing and limb salvage outcomes in patients with postsurgical diabetic foot wounds, who were at high risk of limb amputation [112]. Additionally, Sirvastava et al. also reported six cases of the successful use of Integra for the treatment of intraoral surgical oncologic defects [113]. Photos documenting our experience with Integra are shown in Figure 3.

Another skin substitute Biobrane^®^ is a cell-free, dermal skin substitute made of a nylon collagen mesh and ultra-thin silicone top layer. This substitute is often used as a temporary wound cover and dedicated to the treatment of burns. The method of its use is relatively simple; the nylon matrix strongly binds the elements of the clot, thanks to the dressing adhering to the wound with incomplete skin thickness until the epithelization occurs. In full-thickness wounds, Biobrane should be removed from the wound prior to the skin graft. It can be used to cover burns with incomplete skin thickness, skin donor sites or in skin defects caused by diseases such as epidermolysis bullosa [114]. However, there are some side effects of Biobrane such as an increased risk of infection. There have also been reports of toxic shock syndrome due to the accumulation of exudates beneath [28]. Feng et al. described the use of Biobrane in two female patients. The first had wounds resulting from superficial burns in the abdomen, perineum and thighs. In the second patient, burns involving the thigh, buttock and perineum areas were noted. After using a skin substitute, the wounds healed without complications. Epidermis was observed on the 7th day after treatment [115]. A clinical trial numbered ACTRN12618000245291 began in 2018, comparing different dressings as a treatment for burns in children. Participants will be assigned to groups treated with a standard dressing with silver, a Biobrane substitute or Biobrane along with a regenerative epithelial mixture (RES). Wound closure time, pain level and presence of scarring or itching will be assessed [116].

The next product, OrCel, is made of human allogeneic keratinocytes and fibroblasts obtained from neonatal foreskin. Fibroblasts are seeded onto a sponge of bovine collagen. This substitute provides an environment for the migration of the patient’s cells, being at the same time a source of growth factors and cytokines. However, it is not a substitute for a permanent skin replacement, but is only a biological dressing. It is indicated for the treatment of chronic wounds and donor sites after skin grafts and in patients with blistering epidermal detachment [117]. Still et al. compared the properties of OrCel and BioBrane dressings. The aforementioned subsites were used in burn patients. OrCel allowed faster skinning, wound healing and the formation of less scarring [118]. Santama et al. compared 17 studies related to wound healing in diabetic patients. They found that EpiFix and OrCel showed similar levels of healing, significantly better compared to standard care. The level of adverse effects in the study groups was also low, at a statistically insignificant level [40].

The OASIS Wound Matrix is a skin substitute available as a single or triple layer (OASIS UltraA) product. It consists of an acellular scaffold of the extracellular matrix of porcine jejunum mucosa and is indicated for a wide variety of wounds [95,119]. The Oasis Wound Matrix substitute has been used to treat severe bedsores. Sixty-seven patients were assigned to the group treated with the substitute, while 63 patients were treated with standard treatments. Healing was observed in 40% of patients in the treatment group compared to 29% in the control group. In more than half of the patients with the innovative dressing, the wound size was reduced by 90% [120]. Histopathological samples of tissue obtained from patients treated with Oasis Ultra or standard treatment were also compared. The wounds treated with the substitute showed less inflammation, contracted better, and tissue repair was visible. There were no treatment-related side effects observed [119].

Nevelia is a multilayered skin substitute that consists of a three-dimensional porous matrix of stabilized bovine type I collagen and a semi-permeable silicone membrane that acts as a pseudo-epidermis [121]. A 2019 study evaluated the safety of the Nevelia substitute in 20 burn patients. A dressing was used in the first stage, followed by an intermediate-thickness skin graft. The average healing time was estimated at 55 days. No adverse effects were noted [122]. Angelis et al. compared Nevelia and Integra in people with traumatic wounds. The aforementioned substitutes were treated with autologous epidermal grafts. Healing was observed 2 weeks after the procedure. Epidermis was also noted after 3 weeks. Nevelia showed better epidermal cell proliferation and angiogenesis compared to Integra [123].

## 4. 3D Bioprinting Skin Substitutes

3D bioprinting involves creating complex, three-dimensional structures. To achieve them, several elements are required, such as a bioink composed of cells and a compatible biomaterial. The bioprinting method can be used in tissue engineering, regenerative medicine, cancer treatment, transplantation or drug testing [124].

**Figure 4:**
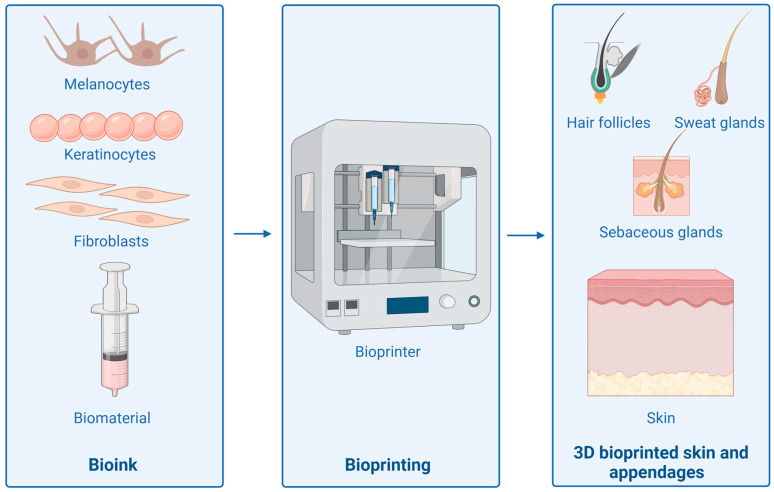
Basic steps during 3D bioprinting of skin and its appendages. Created with Biorender.com (accessed on 5 February 2024).

Skin bioprinting is a complex process with several elements to keep in mind. The manufactured product must have adequate mechanical properties and a functional, semi-permeable layer on the surface. In addition, it is necessary to map the layers of the skin including the cells. The pore size of the construct should be in the range of 200 to 400 µm. The time after which the structure will biodegrade should be at least 3 weeks to allow cell proliferation and blood vessel formation [125]. A scheme of a 3D skin bioprinting process is shown in Figure 4.

Kesevan et al. created a two-layer skin construct composed of plasma, fibroblasts and keratinocytes. These were tested in vitro, and then transplanted into immunodeficient mice. In histological analyses, the bioprinted skin showed properties similar to natural skin and two-layer dressings produced by other techniques [126]. Lian et al. printed a bilayer skin construct composed of a hydrogel composed of gelatin/sodium alginate/gelatin methacrylate and cells—keratinocytes and fibroblasts. Then, they applied it to full-thickness skin lesions in nude mice. The use of the construct allowed for faster epithelialization and healing than when the hydrogel alone was applied. The skin thickness was similar to normal skin. Blood vessel formation was also observed [127]. Alban et al. proposed a new concept for skin bioprinting. It allows for printing at the wound site, which allows for customization of the construct. The model includes the use of both fibroblasts and keratinocytes in full-thickness skin lesions in mice and pigs. The study achieved accelerated wound healing, after which the skin was fully healthy and functional [128]. Levin et al., on the other hand, proposed a prototype of an in situ 3D bioprinter. The bioink was Viscoll, which was combined with endothelial cell lines (HUVEC) and human dermal fibroblasts (HF). The prepared constructs were printed on skin wounds in rats and pigs. The use of substitutes allowed for rapid wound healing, which may be related to various processes such as reduced inflammation, inhibited fibrosis or increased angiogenesis or regeneration [129].

In skin bioprinting, it is also necessary to reconstruct skin appendages such as hair follicles and sebaceous and sweat glands. Abaci et al. used 3D bioprinting to produce hair follicles in skin constructs. The technology enabled the spheroids of dermal papilla cells to form spontaneously in the extracellular matrix. Previous techniques did not allow the reproduction of human hair follicles with such similar properties [130]. Sebaceous gland printing is a less studied area of science. Chen et al. used 3D printing to create a polycaprolactone scaffold, which was then covered with a cell-free matrix derived from adipose tissue-derived mesenchymal stem cells. A line of immortalized human sebaceous cells was seeded onto the constructs. Cell proliferation and secretion of neutral lipids were observed in vivo after implantation under the skin of nude mice. The tested model was biocompatible and did not cause cytotoxicity [131]. Nevertheless, Yao et al. used alginate-gelatin hydrogel along with mesenchymal stem cells (MSCs) as a bioink to 3D bioprint sweat glands in mice. It was proven that the structure of the construct provided better cell proliferation and aggregation. CTHRC1 and Hmox1 were selected as genes required for the differentiation of MSCs into sweat glands [132].

## 5. Immune Reaction to Skin Substitutes

The body’s immediate effect on the substitute is the accumulation of plasma proteins on its surface. Factor XII and tissue factor initiate the coagulation cascade that leads to clot formation. Platelets, monocytes and macrophages adhere to cells using adhesion receptors with accumulated plasma proteins. Cells that adhere begin to secrete growth factors and cytokines that recruit immune cells to the implantation site. The activity of fibroblasts and mesenchymal stem cells allows for the organization of the collagen matrix. The role of macrophages is to migrate towards sites of inflammation. There, they become activated, where they ultimately polarize towards pro-inflammatory M1 and anti-inflammatory M2 states [133].

The role of M1 macrophages includes participation in the phagocytosis of pathogens (destruction and removal of damaged cells, including neutrophils). The M2 phenotype of macrophages is involved in repair and regeneration processes, secretes anti-inflammatory mediators, growth factors and suppressors of cytokine-signalizing proteins. M2 macrophages turn off the pro-inflammatory effect of M1 macrophages and promote the migration and proliferation of fibroblasts, keratinocytes and endothelial cells [134]. Polarization of macrophages from M1 to M2 shows the process of macrophage differentiation, which causes a shift in inflammatory functions to pro-regenerative functions. This change is an important step in the wound healing process. Macrophage polarization from M1 to M2 is activated by IL-4, IL-10, glucocorticoids, prostaglandins, modulators of glucose and lipid metabolism. Macrophage polarization from M1 to M2 can be enhanced by IL-4. Increased numbers of M2 macrophages may cause increased levels of IL-10, IL-12 and TGF-beta. Macrophage polarization occurs in stages and in an ordered manner [135].

When it comes to the implantation of a skin substitute in the inflammatory phase, the presence of M1 macrophages is indicated, but their prolonged presence does not favor the appearance of a foreign body reaction and fibrous encapsulation, which results in the occurrence of chronic inflammation and lack of integration of the substitute [136]. Therefore, the presence of M1 macrophages should be short-lived. Mast cells and basophils activate M2 macrophages, whose task is to activate anti-inflammatory cytokines and support tissue remodeling. Thanks to these actions, it is possible to improve the vascularization of the substitute, thus preventing the formation of fibrous tissue. Sensing signals from the environment allows macrophages to change their polarization. During the healing process, the macrophage phenotype changes from a pro-inflammatory phenotype to a healing-promoting profile [137]. A high ratio of M2:M1 macrophages in the environment of implanted scaffolds ultimately results in better remodeling results. The prolonged presence of M1 macrophages prolongs the inflammatory phase. On the other hand, the prolonged presence of M2 macrophages may result in the formation of detrimental foreign body giant cells [133].

## 6. Conclusions and Future Directions

The enormous progress that has been made in biotechnology, immunology and tissue engineering has resulted in the creation of new advanced skin substitutes for clinical applications. Bioengineering substitutes offer a promising alternative to conventional autologous and allogenic skin grafts and traditional dressings. Chronic wounds and large skin defects require new therapeutic strategies, hence advanced dressings may be the future in the treatment of these conditions. Expanding knowledge about biomaterials, 3D printing and tissue regeneration mechanisms will contribute to the creation of even more advanced skin substitutes in the future. They will not only cover the wound, but also stimulate tissue regeneration, inhibit infections and the formation of hypertrophic scars and keloids. A notable challenge lies in the development of vascularized skin substitutes. Addressing this challenge will require further research, particularly in the development of biomaterials that stimulate angiogenesis, as well as techniques for fostering capillary formation in vitro. The subsequent hurdle will involve integrating these advanced substitutes with the patient’s tissues. However, with the ongoing progress in 3D printing and biotechnology, these types of advanced substitutes may be used clinically in the future.

## Figures and Tables

**Table 1:** Environmental factors in wound pathology.

Factor Type	Cells/Molecules	Role in Wound Pathology	References
Cells	Macrophages	Macrophages show impaired bacterial phagocytosis, removal of apoptotic cells and reduced polarization capacity.	[11]
Cells	Neutrophils	Exhibit cytotoxic activity. They are less susceptible to removal by macrophages and apoptosis.	[12]
Cells	Langerhans cells	Cells with impaired migration in chronic wounds, resulting in reduced epithelialization.	[13]
Metalloproteinases	Serine proteases	Degrade extracellular matrix components and growth factors.	[14]
Metalloproteinases	Collagenases (MMP1, MMP8, MMP13)	Abnormal regulation of inflammation. Overexpression in keratinocytes delays re-epithelialization.	[15]
Metalloproteinases	Gelatinases (MMP2, MMP9)	Secreted by fibroblasts and keratinocytes. Exert an antifibrotic effect.	[16]
Metalloproteinases	Stromelysins (MMP3)	Secreted by keratinocytes. Interferes with wound contraction.	[17]
Growth factors and other	B-cathenin	Inhibition of keratinocyte migration.	[18]
Growth factors and other	c-myc	Inhibition of keratinocyte migration.	[19]
Growth factors and other	TNF-α	Prolong the inflammatory phase, increase metalloproteinase activity.	[20]
Growth factors and other	IL-1	Prolong the inflammatory phase, increase metalloproteinase activity.	[21]
Growth factors and other	IL-6	Interleukin associated with cellular aging and increased inflammatory response.	[22]
Growth factors and other	IL-10	Interleukin associated with phagocytosis.	[23]
Growth factors and other	Maspin	Anti-angiogenic factor secreted by keratinocytes.	[24]

## Data Availability

The work does not contain any additional information or other supporting data.
